# Seeds of trust ripe as luscious fruits: Faculty mentorship forum at Aga Khan University Medical College

**DOI:** 10.12669/pjms.39.5.7175

**Published:** 2023

**Authors:** Rehana Rehman, Rahila Ali, Saira Khalid, Tazeen Saeed Ali

**Affiliations:** 1 Rehana Rehman, Professor, Department of Biological & Biomedical Sciences, Aga Khan University, Karachi. Pakistan; 2 Rahila Ali, Senior Instructor, Department for Educational Development, Aga Khan University, Karachi. Pakistan; 3 Saira Khalid, Nursing Instructor, College of Nursing Armed Forces Postgraduate, Medical Institute (AFPGMI), Rawalpindi. Pakistan; 4 Tazeen Saeed Ali Interim Dean, School of nursing and Midwifery, Aga Khan University, Karachi. Pakistan

**Keywords:** Mentorship, Mentors, Mentees

## Abstract

**Background & Objective:**

Faculty members require mentoring in all stages of their professional development. Aga Khan University (AKU) has initiated mentorship programs for students and faculty at AKU Medical College (AKU- MC) and AKU School of Nursing and Midwifery (AKU-SONAM). This study aimed to explore perceptions of mentors, mentees, administrators (chairs, co-chairs, founder members and coordinators of the mentoring program) and leadership and further investigate the strengths and challenges faced by ‘Faculty Mentorship Program’ at AKU- MC.

**Methods:**

We conducted a qualitative exploratory study from February till December 2021 after approval from AKU using purposive criterion sampling. Mentors, mentees, administrators and dean of AKU- MC were included in the study. The interview guide was developed, validated and reviewed by experts. After piloting, four focused group discussions and 8 in-depth interviews were conducted in AKU- MC.

**Results:**

The findings described the transition from informal to formal mentorship, identified challenges faced by mentors and mentees and suggested the role of leadership / administrators. The themes identified were “Continuous improvement of mentoring practices”, “Building strong foundations for mentoring relationships”, and “Growth and development through challenging experiences”

**Conclusion:**

The faculty mentorship program at AKU-MC was in line with the goals and vision of the institution. All participants identified the need of additional resources, administrative support, rewards, incentives and recognition of mentors for sustainability of the program.

## INTRODUCTION

Mentorship persists as a relationship between two individuals usually a senior and a junior for focused and proficient development of the later.^1^ It exists as: “A process whereby an experienced, highly regarded, empathetic person (the mentor) guides another (usually younger) individual (the mentee) in the development and re-examination of their ideas, learning, and personal and professional development. The mentor, who often (but not necessarily) works in the same organization or field as the mentee, achieves this by listening and/or talking in confidence to the mentee”.^2^

The support by a mentor is provided informally or through an organized systematic plan.^3^ In addition to student mentoring, faculty mentoring is essential for personal and professional development of faculty and reported to improve productivity, promotion, and retention rates.^4^ The relationship is beneficial to mentees as well as the mentors in research, education and health care.^5^ Mentorship program at AKU Medical College (AKU-MC) is looked after by “Faculty Mentorship Forum (FMF)”. The forum evolved in 2019, after the initial work performed by Faculty Mentorship Task force committee (April 2014 to December 2014) and standing committee from 2014 to 2018.^6^

There is a dire need to institute faculty mentorship programs to ensure personal development, provide career guidance and monitor academic productivity of mentees through a structured format.^7^ It is also required to monitor processes, strengths and weaknesses of these programs on a continuing basis and report the outcomes.^8^ The study aimed to explore perceptions of faculty (mentor, mentee), administrators and leadership about faculty mentorship program and examine its strengths and challenges.

## METHODS

A qualitative exploratory study was carried out from February till December 2021 following endorsement from ‘Ethical Review Committee of the Aga Khan University, Karachi: (2021-6127-17832)’. Mentors, mentees, administrators and leadership were recruited by purposive sampling technique. Mentors and mentees in FMF for at least one year with participation in at least two mentoring sessions were included. Chairs, co-chairs, founder members and coordinators of the FMP with at least one-year service were counted as administrators. Leadership comprised of Dean of AKU-MC and Associate Dean Faculty AKU-MC. We aimed to include leadership and administrators for in depth interview (IDIs) to capture their unique experiences and perspectives, allowing for a deeper exploration and providing confidentiality.

However, focus groups were chosen for mentors and mentees to gather a range of perspectives allowing a more comprehensive understanding and encouraging discussion for a deeper exploration. With the help of relevant literature, the researchers formulated a semi-structured guide with open ended, introductory, probing and concluding questions for both IDIs and focus group discussion (FGDs). The guide underwent Delphi rounds of review by experts selected on the basis of their experience in mentoring and qualitative studies. Both interview guides were piloted after which interviews were conducted in a private setting ensuring a comfortable environment. All the interviews were video recorded, and transcribed. Participants were given a chance to review the transcript to confirm accuracy which helped in avoiding bias (Member Check). In order to control biases, the researcher maintained reflective logs after the IDIs and FGDs regarding the perceptions about mentorship program. The reflective logs were shared with thesis committee members and supervisor to maintain the objectivity of data as well as to minimize personal biases. Data collections like IDIs, FGDs were triangulated with Document review. Data collected from different study groups such as leadership, administrators, mentors and mentees was triangulated amongst themselves. The triangulation of results of the researcher, investigators and committee members (investigators triangulation) was carried out simultaneously to enhance validity, credibility and rigor of the study.

For the analysis of data, codes were developed by the authors independently to maintain trustworthiness. These codes were then shared in a meeting with the committee members where a consensus was obtained to finalize them. Similar codes were combined together, grouped into sub-categories and categories to identify and then finalize the respective themes.^9^,^10^

## RESULTS

**Fig.1:**
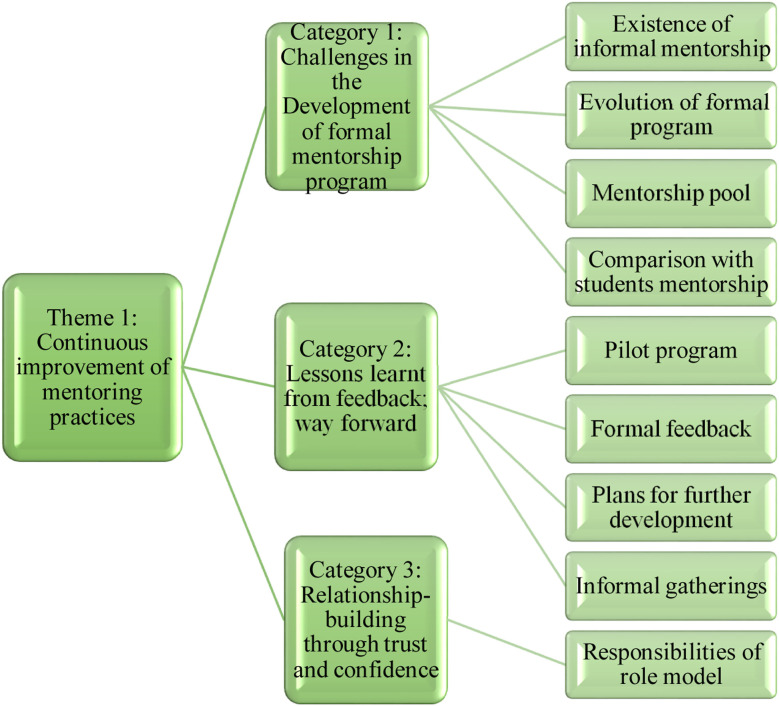
Theme-1: Continuous improvement of mentoring practices

Seventeen individuals from AKU-MC (six administrators, two leaders, four mentors and five mentees) participated in the study (Table-I; supplementary data). Eight IDIs were conducted with administrators and leadership, two FGDs were conducted with mentors with two participants in each whereas two FGD for mentees comprised of two and three participants. The codes and categories of first theme ‘Continuous improvement of mentoring practices” elucidated the elaboration of AKU-MC mentorship program on the basis of following three categories (Fig.1).

**Table-I:** Demograhics of Study Participants (Supplementary Data)

Characteristics of informants in IDIs at AKU-MC			

IDI No.	Gender	Position/Status/Role	Association with Mentorship Committee and Forum
IDI 1	Female	Founder member	5 years
IDI 2	Male	Founder member	5 years
IDI 3	Female	Chair Mentorship Forum	5 years
IDI 4	Female	Administrator	3 years
IDI 5	Male	Leadership	4 years
IDI 6	Male	Leadership	2.5 years
IDI 7	Female	Founder member	4 years
IDI 8	Male	Member	3 years

***Characteristics of informants (mentors) in FGD 1, AKUMC***			

*Informants*	*Gender*	*Position/Status/Role*	*Duration of attachment with the institution*

P1	Female	Associate Professor	More than 10 years
P 2	Female	Professor	20 years
P3	Female	Professor	22 years
P4	Female	Assistant Professor	6 years

***Characteristics of informants (mentees) in FGD 2, AKUMC***			

*Informants*	*Gender*	*Position/Status/Role*	*Duration of attachment with the institution*

P 1	Male	Assistant Professor	2 years
P2	Female	Assistant Professor	3 years
P3	Male	Resident	5 years
P4	Female	Senior Instructor	5 years
P5	Male	Senior Instructor	3 years

Category ‘Challenges in the Development of formal mentorship program’ explained evolution of formal program as; “In 2014, the Dean formulated a committee which presented a report on the need of mentorship programs. The findings assisted the formation of a formal committee that worked from March 2017 till June 2019 from June 2019 onwards, name of committee was changed to Faculty Mentorship Forum” described in an IDI with one of the administrators. A mentor added; “Informal mentorship existed a couple of years back, before the introduction of formal mentorship program. The mentees used to work with mentors of their own department who helped them to achieve desired goal”.

**Fig.2:**
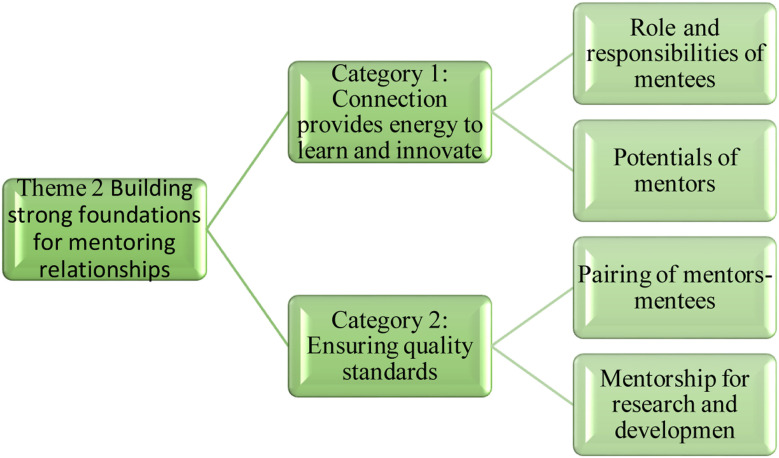
Theme-2: Building strong foundations for mentoring relationships

Importance of formal feedback was highlighted in the category of ‘Lessons learnt from feedback; way forward’. All participants recommended the importance of documentation of all mentorship activities with emphasis to develop guidelines, handbook and mentorship curriculum. A mentee emphasized, “I think there should be slightly more structured and formal role of university to document the process and outcomes”. In the plans for further development participants highlighted the importance of informal gatherings where mentee could deliberate their doubts, sorrows and concerns with their mentor. A mentor mentioned, “There should be breakfast and tea meetings between new and old mentors at regular intervals so that they could share their experiences. This way the new mentors will learn the traits of a previous role model” (IDI-02). The category of: “Relationship-building through trust and confidence “accentuated the responsibilities of Role Models One mentor shared his experience in terms of, “I remember my mentor would never leave the lecture hall before erasing the whiteboard / chalkboard for the next person. This small gesture sets a path. There are so many lessons for young students who can learn from a simple piece of role modelling”. Theme “Building strong foundations for mentoring relationships” (Fig.2) discusses role and responsibilities of mentors and mentees to maintain quality standards.

**Fig.3:**
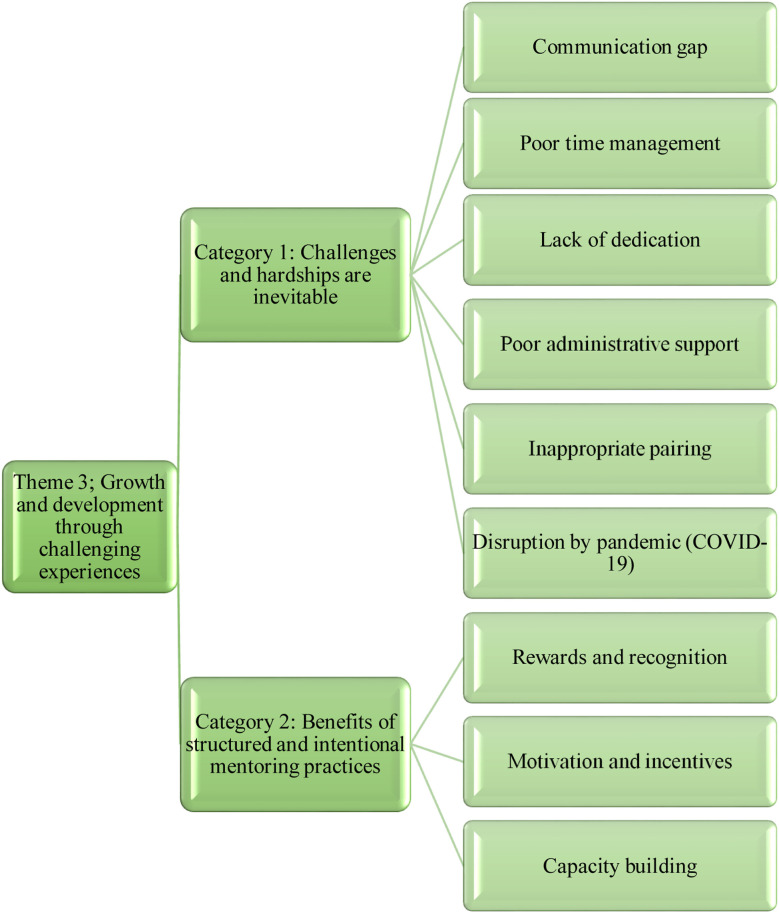
Theme-3: Growth and development through challenging experiences.

In its category of ‘Connection provides energy to learn and innovate’, (code; Role and Responsibilities of Mentees), one mentee shared, “I think a good mentee should try to find means to be in touch with their mentor especially when the mentor is extremely busy and not available on emails”. An administrator mentioned; “The list of mentors is available on website which is reviewed every two years. FMF follow a Dyad Model of Mentorship^11^; match junior faculty with available mentors. The mentees are being provided with option to come back if they are unhappy or unsatisfied with it, they can request to change their mentors”. Mentorship for research was considered important as mentioned by an administrator “The research office provides mentorship and guidance to all the faculty members through the senior leadership forum” (IDI-06). Theme-III: Growth and development through challenging experiences is illustrated in Fig.3

In this theme all the barriers, challenges like lack of commitment and effective communication between both mentors and mentees, limited resources such as time, budget, and administrative support required for sustainability of the program was highlighted. Improper pairing with limited access to mentors was also an additional issue identified in this study. In the category of ‘Challenges and hardships are inevitable’, communication gap was identified as the biggest challenge faced by mentors and mentees.

Mentees mentioned the difficulty in scheduling of meetings whereas mentors mentioned lack of protected time as one of the biggest challenges. A mentee verbalized; “Many young faculty members were facing difficulty to interact with some of the senior faculty members. The culture of face to face communication has to be revived instead of exchanging emails or WhatsApp messages. A solution to improve the communication gap is participation in departmental and coffee meetings.” (FGD-01 P-03). Both mentors and mentees recognized the importance of time management to overcome all the hurdles. One mentor commented, “The first year of my mentorship, I had two mentees. I could never meet one mentee for the whole year and later the mentee went for some more training. I had about three good and interesting meetings with the second mentee”. (FGD-01 P-03)

In the category of ‘Benefits of structured and intentional mentoring practices’, participants emphasized the importance of celebrations to recognize and reward the faculty at departmental as well institutional levels. An administrator stated, “The awareness and recognition of program needs attention. It is important to organize awareness sessions as well as disseminate success stories. Support group on education can help in coordination of activities. Mentors should be recognized in faculty assemblies by announcing their names. There should be university culture of awards and recognition” (IDI-01).

Mentees strongly recommended that higher authorities should appreciate and motivate the mentors. A mentee added, “There should definitely be some areas or may be some components of support from the administration to appreciate the mentors. There should be a mechanism to recognize the mentors officially. There is a need to acknowledge mentoring, market and scale up the program and share successes stories so that other faculty members realize the benefits of the program”.

## DISCUSSION

The study explored the importance of faculty mentoring in promoting the personal and professional growth of mentees through effective communication, skill sharing, and role modeling. The study highlighted the prevalence of the dyad mentorship model, which pairs proficient mentors with junior mentees, and emphasized the importance of open communication and time commitment from both mentors and mentees. The availability of a mentor pool with volunteers of varying qualifications, trainings, and experiences was identified as a successful measure in our study. Mentors obtained ample opportunities to connect, share ideas and strategies, cultivate practical skills, and broaden perspectives that lead to personal growth and career progression of mentees which is supported by literature.^12^

There are a number of mentoring models which support mentors to deliberate cognitive growth and personality development of mentees.^13^ Regardless of the model, the relationship is constructed by provision of opportunities to both mentors and mentees for an effective communication.^14^ Dyad mentorship model which is the most prevalent model was practiced in our mentorship program where pairing of proficient mentors with junior mentee was strengthened by FMF.^11^

In depth exploration revealed that mentors enrolled in the program were eager to learn, transfer their wisdom to juniors and guide them in the career path. Literature also supports role of mentors in growth and development of mentees and students.^15^ The interviews also recognized the importance of role models as a source of inspiration in education, teaching and research whose guidance prepared their recognition and career development. A mentor also familiarizes the mentee about the learning environment and the career pathway and facilitates as a role model in good practices and professional behaviors.^16^,^17^

The study emphasized the importance of open communication and time commitment equally important for both mentors and mentees. Respect for mentees with positive feedback with sharing of relevant resources like guidelines, research papers and reading material enhance progressive prospects and promote career growth of mentees.^18^ Our study highlighted the availability of a mentor’s pool that comprised of volunteers with different qualifications, trainings and experiences with a baseline criterion. Furthermore, the mentees were given the choice to select the mentors. The accessibility of mentor’s pool is deliberated as a successful measure in many studies.^19^.^20^ Our participants highlighted the responsibility of both the, mentors and mentee to make possible efforts to strengthen the working relationship. Moreover, it is also important that meeting time should be scheduled after careful consideration of mentor’s schedule.

The study also emphasized the importance of institutional support in preparing mentor-mentee meetings, conducting evaluations, and improving programs based on obtained feedback. It recommended the use of online mentoring and providing flexibility to individuals from all backgrounds who wish to participate in mentorship programs. The need to support consistency and permanence, mentorship guidelines with well-defined objectives, clear expectancies, and description of roles and responsibilities of mentors and mentees was also identified 21. After setting the guidelines monitoring and evaluation of all the processes and outcome for evaluation of impact decisions and policy making also came through the discussions.^22^,^23^ It was also recommended to make use of online mentoring and provide flexibility to all those who wish to get mentored irrespective of gender, ethnicity, disability and geographical location.^24^

### Strengths of the study

This is the first ever study conducted in Pakistan to evaluate the mentorship program in the health care system. The study achieved a high level of success in gathering comprehensive information from diverse hierarchical positions, enabling a range of perspectives and recommendations for improvement. The researcher’s involvement in the entire process of data collection and analysis contributed to the study’s strength and credibility. The rigor was further enhanced through data triangulation involving the researcher, research coordinator, and committee members. The information obtained by the in-depth exploration could be useful for healthcare organizations and institutions to design and implement mentorship programs for their faculty and enhance their personal and professional development

### Limitations of the Study

To gain a comprehensive understanding, a detailed and systematic collection and analysis of the program’s proposal, process, and implementation is necessary, which was beyond the scope of this study. Furthermore, the limited duration of the study made it challenging to assess both the short-term and long-term outcomes of the program, including proximal, enabling, and distal outcomes. However, the study provided preliminary insights into the effectiveness of the mentoring program and highlighted areas of improvement

## CONCLUSION

The study interpreted alignment of FMF with vision and mission of AKU-MC representing effective formal mentoring practice which emerged from traditional informal mentoring. The forum followed Dyad mentoring model, organized mentor mentee pairing and followed the relationship with structured feedback system. The participants recognized the need of protected time, strong administrative support and continuation of recognition, rewards and incentives for the mentors.

### Authors’ Contribution:

**RR:** Designed the study.

**TSA** and **RA:** Supervised the whole project.

**RR and SK:** Executed the study under supervision of STA and RA.

**RR:** Is accountable for the accuracy and integrity of the study.

All authors read the manuscript and revised the content.
